# Implementation of postpartum navigation for low-income individuals at an urban academic medical center

**DOI:** 10.1371/journal.pone.0282048

**Published:** 2023-02-23

**Authors:** Hannah M. Green, Viridiana Carmona-Barrera, Laura Diaz, Chen Yeh, Brittney Williams, Ka’Derricka Davis, Michelle A. Kominiarek, Joe Feinglass, William A. Grobman, Chloe Zera, Lynn M. Yee

**Affiliations:** 1 Division of Maternal-Fetal Medicine, Department of Obstetrics and Gynecology, Northwestern University Feinberg School of Medicine, Chicago, IL, United States of America; 2 Biostatistics Collaboration Center, Department of Preventive Medicine, Northwestern University Feinberg School of Medicine, Chicago, IL, United States of America; 3 Division of General Internal Medicine and Geriatrics, Department of Medicine and Preventive Medicine, Northwestern University Feinberg School of Medicine, Chicago, IL, United States of America; 4 Division of Maternal-Fetal Medicine, Department of Obstetrics and Gynecology, The Ohio State University, Columbus, OH, United States of America; 5 Division of Maternal-Fetal Medicine, Department of Obstetrics and Gynecology, Beth Israel Deaconess Medical Center, Boston, MA, United States of America; Universiteit Twente, NETHERLANDS

## Abstract

**Background:**

Patient navigation, a patient-centered intervention to promote comprehensive health care, is an emerging innovation in obstetrics to optimize postpartum care. We aimed to evaluate the implementation of a novel postpartum patient navigation program at an urban academic medical center.

**Methods:**

This mixed-methods study analyzed the implementation of a postpartum patient navigation program within an ongoing randomized control trial. This study analyzed three navigators’ logs of interactions with 50 patients, care team members, and community organizations throughout patients’ first year postpartum. We categorized and quantified interactions by topic addressed, care team member interacted with, and communication mode used. We also conducted semi-structured interviews with each navigator every three months (5 interviews per navigator), emphasizing navigation experiences, relationships with patients and care teams, integration in the care team, and healthcare system gaps. Interview data were analyzed using the constant comparative method to identify themes using the constructs of the Consolidated Framework for Implementation Research (CFIR).

**Results:**

Analysis of navigator logs revealed a high patient need level, especially in the first 3 months postpartum. CFIR-guided analysis of intervention characteristics revealed positive perceptions of navigation’s utility due to its adaptability. Navigation’s complexity, however, posed an early obstacle to implementation that diminished over time. Outer setting analysis indicated navigators addressed patient needs through interactions with multiple systems. Despite clinicians’ initial unfamiliarity with navigation, inner setting analysis suggested ongoing communication and electronic medical record use facilitated integration into the care team. Regarding individual and process characteristics, findings emphasized how navigator self-efficacy and confidence increased with experience (individual) and was facilitated by comprehensive training and reflection (process). Overall, barriers to implementation included unfamiliarity, varied patient engagement, and innovation complexity. Facilitators included high patient need, communication with outside organizations, medical record usage, navigator characteristics (self-efficacy, communication skills, and personal growth), a comprehensive training period, consistent reflection, high relative advantage, and high adaptability to patient need.

**Conclusion:**

Patient navigation is a promising innovation to improve postpartum care coordination and support care team efforts. The successful implementation of navigation in this study indicates that, if shown to improve patient outcomes, obstetric navigation could be a component of patient-centered postpartum care.

## Introduction

As periods of consistent and even increased access to care, pregnancy and the postpartum year offer unique opportunities to optimize the future health of birthing individuals [1–5]. Moreover, high patient motivation increases engagement with obstetric and postpartum care [6]. Despite this potential opportunity to improve outcomes, disparities in postpartum care persist, particularly among individuals with low incomes, resulting in inequitable health outcomes [7, 8]. One reason for the inequity is the complexity and fragmentation of the United States health care system, which threatens the longitudinal health of birthing individuals. For example, challenges in successful patient transitions from postpartum to primary care can be exacerbated by chronic illness and the need for specialty care [5]. Care coordination and interdisciplinary collaboration can facilitate a smooth handoff between care teams [9, 10], yet even for medically uncomplicated individuals, obstetric and primary care providers have identified multilevel barriers to this transition [11].

Patient navigation, a patient-centered, individualized intervention designed to identify and address barriers to care, was first implemented in oncology to ease burdens of fragmented care [12], but has since been successfully incorporated into diverse health settings [13–15], including postpartum care [16, 17]. By identifying and mitigating individual barriers to accessing care, facilitating care coordination, and providing health education and social support, the use of patient navigators may be a scalable way to optimize postpartum care and improve self-efficacy, thereby improving long-term health care utilization and health outcomes among birthing individuals [17]. However, the interdisciplinary and multilevel nature of navigation, which operates at the individual, clinical, and health systems levels, contributes to the complexity of this innovation [18]. Although clinical outcomes of navigation programs in several settings have been reported, implementation has not yet been widely assessed in postpartum care. As such, identifying facilitators of and barriers to the implementation of postpartum patient navigation is necessary to realize the potential of this innovation in alleviating postpartum disparities and improving health.

This study aims to identify facilitators of and barriers to the activities of postpartum patient navigators and their integration into the medical care team at an urban academic medical center as they navigate patients through one year postpartum. Such identification will serve useful in guiding future implementation of postpartum navigation at other institutions.

## Methods

This is a mixed methods analysis of implementation data within an ongoing randomized controlled trial (RCT) of postpartum patient navigation for low-income individuals, defined as receiving Medicaid-funded prenatal care. In this RCT, Navigating New Motherhood 2 (NNM2), eligible pregnant and postpartum individuals who consent to participation are enrolled as an outpatient (after 30 weeks of gestation) or prior to hospital discharge following delivery and randomized to receive either navigation or usual care for one year after birth. Individuals randomized to navigation receive personalized support from lay individuals trained as postpartum navigators. This tailored support includes activities such as care coordination, health education and health behavior support, communication with medical teams, and individualized attention to social determinants of health that may pose challenges to postpartum health and health care. Over four years, 400 pregnant or postpartum individuals will be enrolled to assess whether postpartum navigation improves clinical outcomes such as retention in care, receipt of contraception, and breastfeeding initiation, as compared to usual care (NCT03922334) [19].

**Fig 1:**
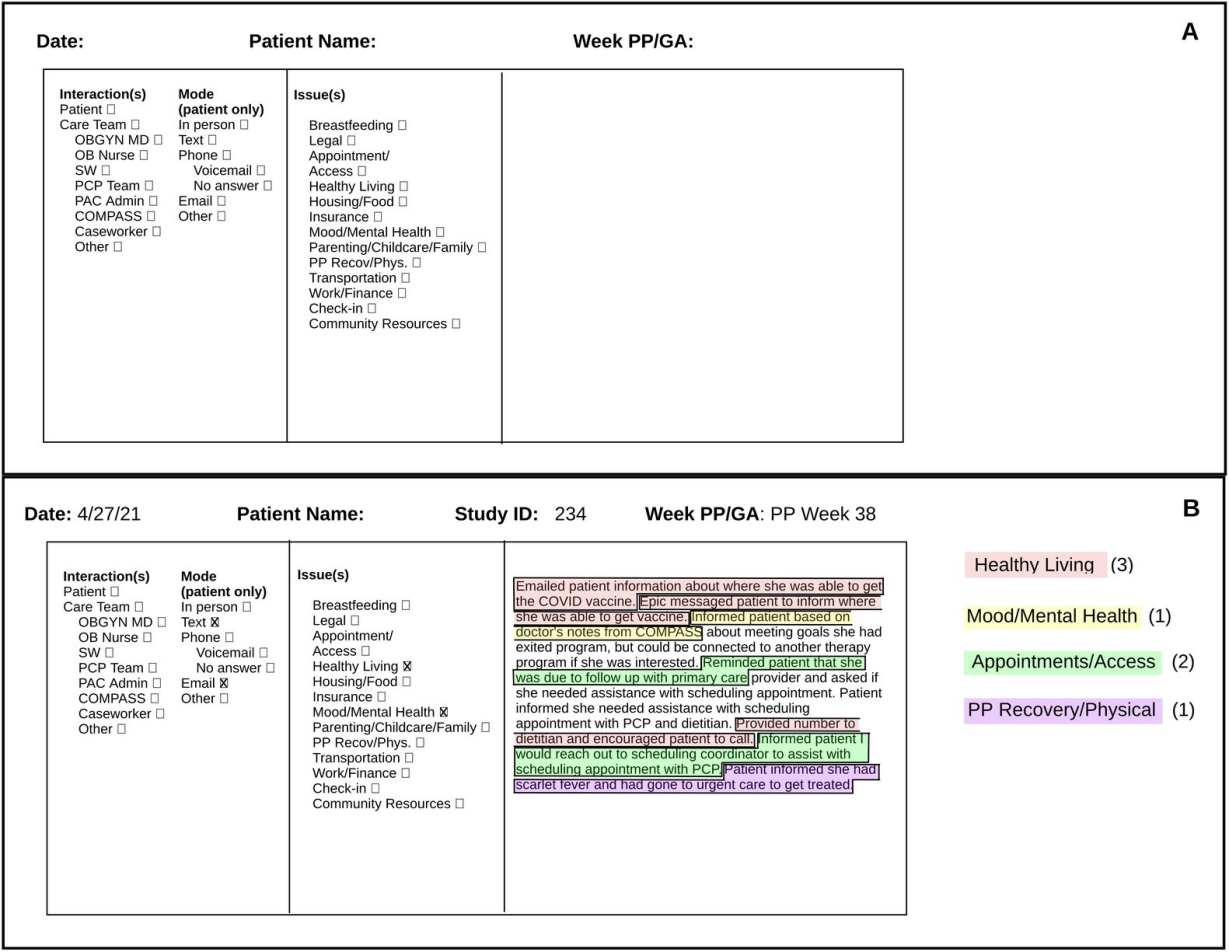
Standardized weekly log used to track navigation activities. A. Empty entry illustrating format of standardized navigator log. Navigators tracked all interactions with patients and care team members, including mode of communication and issue addressed. Navigators also recorded notes to narrate tasks and interactions. B. Sample entry with coding and quantification of themes regarding patient need categories. Notes were coded based on issue addressed, quantified, and entered into REDCap software for data management.

This pre-planned analysis assessed the implementation of navigation for the first 50 individuals randomized to the navigation arm of NNM2. We had an *a priori* goal of analyzing implementation data when the log and interview data (described below) demonstrated saturation, which all investigators agreed had occurred after the first 50 participants. During the trial, navigators had both scheduled (based on postpartum month) and unscheduled (e.g., appointment reminders or patient-initiated communication) interactions with patients to address needs and barriers to care as they arose. They also used the electronic medical record to facilitate communication with the healthcare team and support appointment scheduling and attendance. To track navigators’ activities, we designed a standardized log for navigators to record, in real time, interactions with participants, care team members, and community organizations, as well as other miscellaneous tasks they performed (Fig 1, Panel A). Navigators recorded the mode of communication and content of each interaction. Log data were then abstracted by the research team for evaluation. To quantify navigator tasks, descriptive notes in logs were evaluated verbatim and coded based on the clinical or social issue addressed as listed in the log (Fig 1, Panel B). This process of qualitative analysis of free text was carried out by a content analysis method. Activities addressing patient issues were grouped into one of 5 overarching patient need categories: appointments and access (consisting of issues ’appointment/access,’ ‘insurance,’ and ‘transportation’), parenting (‘breastfeeding,’ ‘legal,’ and ‘parenting/childcare/family’), social determinants of health (‘housing/food’ and ‘work/finance’), health and wellness (‘healthy living,’ ‘mood/mental health,’ and ‘postpartum recovery/physical), and relationship building between navigator and participant (‘check-in’). Activities addressing ‘community resources’ were grouped into the appropriate category based on patient need addressed. Each separate navigator activity such as contacting the patient, contacting the care team, or coordinating a resource constituted one navigator task. Log data were quantified and entered into a data management software, REDCap [20], stratified by participant and postpartum week. Data entries quantified the care team member interacted with, the mode of communication used, and the patient need category each activity addressed (Fig 1, Panel B). This detailed extraction of log data allowed for the quantification of tasks to yield an overall workload (interactions per patient per month) for a postpartum navigator.

Additionally, we conducted semi-structured in-depth individual interviews with each navigator every 3 months (5 interviews per navigator), utilizing appropriate reporting standards [21]. The interview guide, which was developed via team discussion, is available as S1 Appendix. Interviews were conducted by the primary author, who had no prior relationship to navigators. There were two primary navigators for NNM2, as well as a temporary study navigator who participated in a single interview, resulting in 11 total interviews available for analysis. All navigators identified as women; the primary navigators identified as Hispanic/Latinx and were bilingual (English and Spanish). None of the navigators were healthcare professionals although all underwent extensive training to become postpartum navigators [22]. Interviews were audio recorded, lasted approximately one hour, and focused on navigator experiences including relationships with participants, relationships with care team members, integration into the care team, and healthcare systems gaps. We transcribed each interview verbatim and utilized Dedoose (www.dedoose.com), a qualitative data management and analysis software, to code transcripts using the constant comparative method. The first set of interviews yielded an initial codebook, which was shared with and edited by the research team. This codebook was used for subsequent interview transcripts, and this process led to consolidation and refinement of themes via a team-based, iterative process. To ensure participant checking, the navigators themselves participated in the team discussions of codes but all formal coding was done by non-navigator research staff. We then conducted a second round of coding for each transcript using themes based on constructs from the Consolidated Framework for Implementation Research (CFIR) to specifically identify implementation characteristics [23].

The mixed methods evaluation consisted of integrated analysis of both log and interview data using the CFIR framework, which systematically identifies facilitators of and barriers to the multilevel implementation of a novel intervention [24]. First, total navigator tasks from log data were summed to yield navigator workload per postpartum month per participant, illustrating variations in the innovation’s intensity over time. Interactions with care team members were stratified by postpartum month and team member with whom interactions occurred: physician (OBGYN), nurse (OBGYN), social worker, obstetric clinic administration, or primary care team (any team member). Interactions were also separated based on mode of communication by postpartum month to identify mode of navigator communication with participants over time. Qualitatively, themes that emerged from interviews with navigators were analyzed based on the five CFIR domains: intervention characteristics, outer setting, inner setting, characteristics of individuals, and process [20]. Interview quotations pertinent to each domain, based on the construct coded to each quotation, were evaluated to assess implementation. Constructs that both facilitated and hindered implementation were identified.

The NNM2 study was approved by the Northwestern University Institutional Review Board. The NNM2 team received signed consent forms for all enrolled patient participants, and we received verbal consent from navigators at the start of each interview.

## Results

We analyzed navigation implementation for the first 50 participants who were enrolled in NNM2 and randomized to receive navigation. Most participants self-identified as non-Hispanic Black (56%), and 42% self-identified as Hispanic/Latinx. A majority disclosed a household income less than $25,000 per year (64%), and 50% reported they were unemployed. Additional demographic information is provided in Table 1.

**Table 1:** Demographic characteristics of 50 navigated participants.

Demographic	Value	Frequency^1^	%
**Race**	American Indian/Alaskan Native	0	0
	Asian	1	2
	Native Hawaiian or Other Pacific Islander	1	2
	Black	28	56
	White	8	16
	Other/Declined to answer	18	36
**Hispanic/Latinx **	Yes	21	42
	No	29	58
**Household Income **	Under $10,000	16	32
	$10,000-$25,000	16	32
	$25,001-$50,000	6	12
	$50,001-$100,000	2	4
	Don’t Know	9	18
	Declined to answer	1	2
**Education Level **	Some high school or less	3	6
	High school graduate	16	32
	Associate degree or some college	18	36
	College graduate	8	16
	Graduate degree or greater	2	4
	Other	3	6
**Relationship Status**	Single/Unpartnered	20	40
	Living with a partner	21	42
	Married	8	16
	Other	1	2
**Current Work Situation **	Unemployed	25	50
	Part time or temporary work	8	16
	Full time work	10	20
	Student	4	8
	Other	3	6
**Total Pregnancies (Including navigated)**	1	9	18
	2	16	32
	3 or more	25	50

Demographic information for all participants (N = 50), including frequency and percentage in each category. All demographic data were collected at first participant interview with the RCT team and represent self-identified information from participants.

1. Frequencies in race category sum to above 50 due to ability to check multiple identifiers.

**Fig 2:**
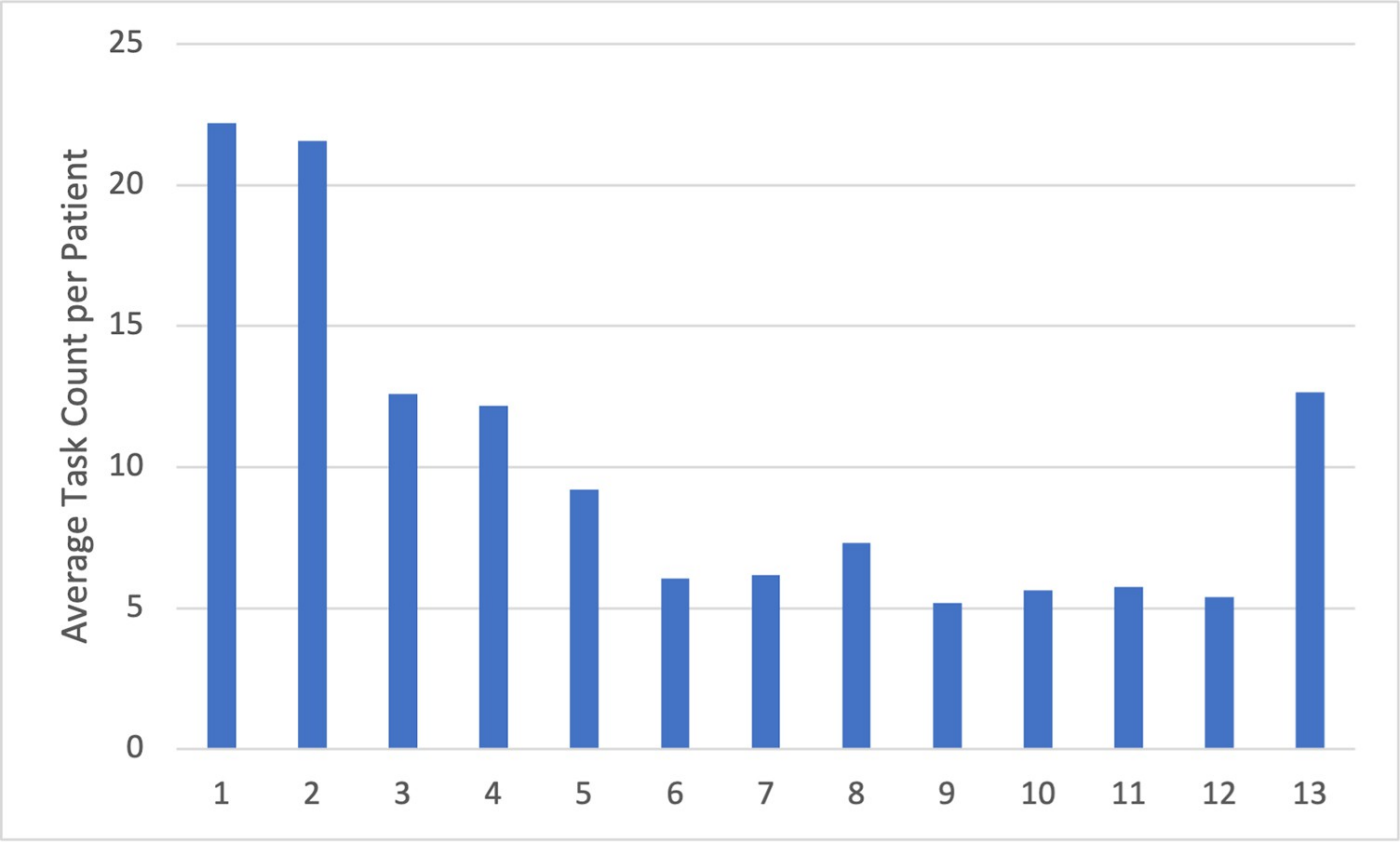
Average navigator workload per participant. Total navigator tasks in each postpartum month were summed across all participants (N = 50). Average navigator workload was calculated by dividing each sum by the number of participants, yielding an average navigator workload per participant per postpartum month.

Analysis of navigator logs revealed a high level of participant need for navigation services in the first 2 months postpartum, with an average number of tasks per patient of 22.2 and 21.6 for postpartum months 1 and 2, respectively. Thereafter, navigator workload tapered throughout the postpartum year, but rose once again approaching the end of the first year, when navigators prepared participants to exit the study (Fig 2). Similarly, the number of navigator interactions with care team members per participant was highest in postpartum months 1 and 2. Given that fluctuations in navigator task load occur as a result of unscheduled, largely patient-initiated contact, these differences represent time-dependent fluctuations in overall patient need, with the highest need level early the postpartum year (Fig 2). Regarding navigator interactions with healthcare team members, the number of interactions with obstetric care team members decreased throughout the postpartum year, while interactions with primary care team members became more common as patients transitioned to long-term care (Table 2).

**Table 2:** Total number of navigator interactions with care team members by postpartum month.

	Postpartum Month												
Care Team Member	1	2	3	4	5	6	7	8	9	10	11	12	13
Physician (OBGYN)	54	69	11	20	13	3	7	4	4	7	2	0	1
Nurse (OBGYN)	43	8	6	2	6	2	0	2	1	0	1	1	0
Social Worker	40	27	10	2	6	4	0	4	2	2	8	1	0
Obstetric Clinic Administration^1^	27	24	6	10	9	3	6	3	2	1	4	0	2
Primary Care Team^2^	1	15	10	11	9	6	2	14	10	12	6	20	16
Total	165	143	43	45	43	18	15	27	19	22	21	22	19

Navigators recorded all interactions with care team members using a standardized weekly log. Interactions with each care team member were quantified, stratified by participant and postpartum month. Data represent total navigator interactions with care team members for all participants (N = 50) for each postpartum month.

1. Obstetric Clinic Administration includes patient service representatives and scheduling coordinators.

2. Primary Care Team includes scheduling coordinators, primary care physicians, and primary care nurses.

The majority of navigators’ communication with participants occurred via text message, ranging from 64.1% of all communications in postpartum month 1 to 83.6% of all communications in postpartum month 11 (S1 Table). In-person interactions between navigators and participants were most common during the initial and final months of the postpartum year due to the nature of clinical care and research study visits, which included navigation exit activities.

Analysis of descriptive notes in navigator log entries illustrated the complexity of navigation activities. Narrative accounts of log entries recounted multiple tasks to address patient needs. For example, an entry which recounted one navigator’s attempts to assist a patient with breastfeeding support contained tasks including providing the patient with information regarding multiple resources, calling resources directly, motivating the patient to access resources as needed, and following up with the patient repeatedly. These complex interactions addressed patient needs but required intense navigator work that could span multiple days. High-task log entries were more prevalent for participants who regularly engaged in navigation, as demonstrated through a pattern of high levels of communication between patient and navigator, indicating navigation’s potentially increased benefit for high-needs patients with willingness to engage in care initiatives. Further, the content of log entries varied widely, demonstrating the breadth of navigation activities.

**Fig 3:**
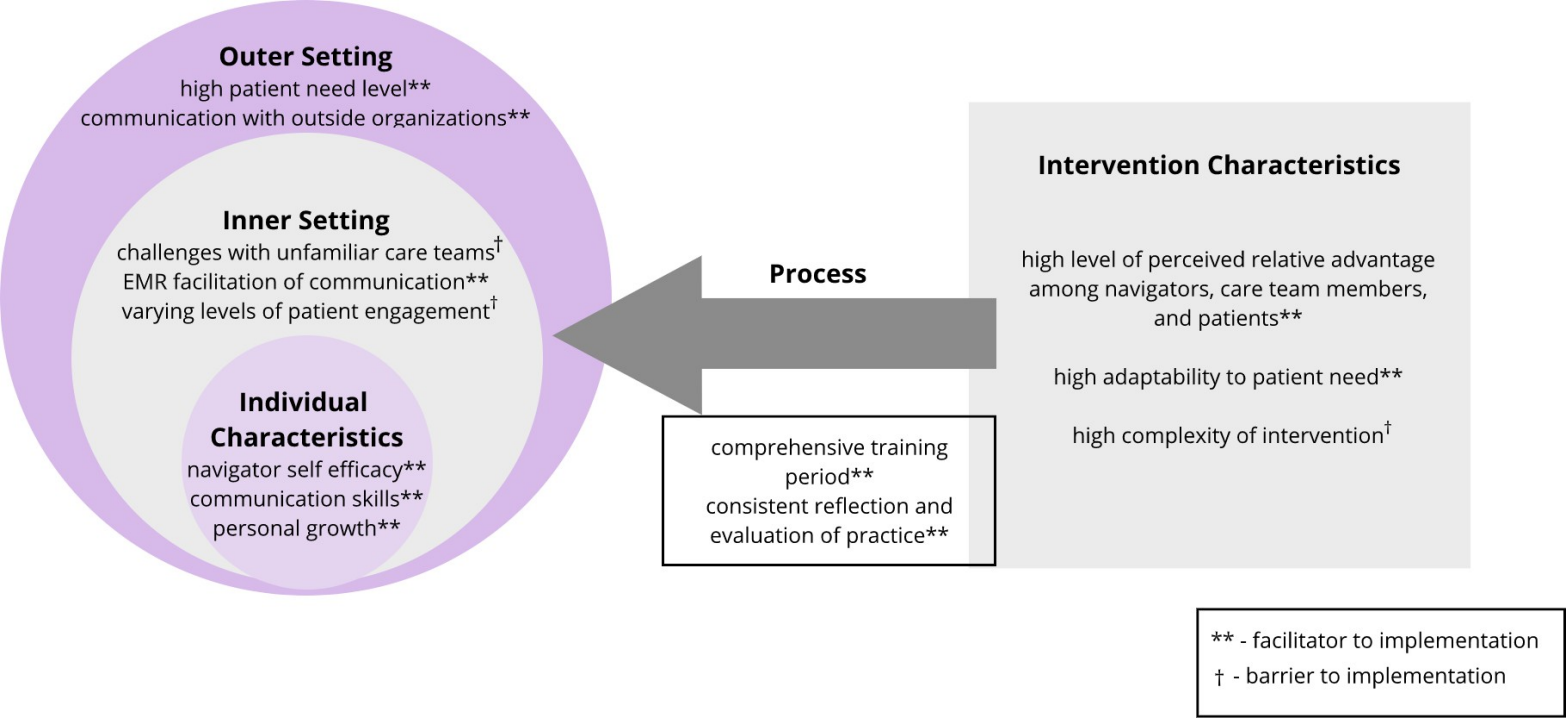
CFIR-guided analysis of navigation implementation. Analysis of log and interview data yielded themes across all five domains of the Consolidated Framework of Implementation Research (CFIR). Qualitative coding was conducted using the constant comparative method to identify themes using CFIR constructs. CFIR constructs were synthesized to identify facilitators of and barriers to the implementation of navigation were mapped to CFIR domains.

CFIR-guided analysis of interviews with navigators revealed facilitators of and barriers to implementation across all 5 CFIR domains (Table 3, Fig 3). CFIR constructs within each domain are italicized. Fig 3 interprets the findings using study-specific themes that are mapped to the CFIR constructs, as shown in Table 3.

**Table 3:** Themes from navigator interviews regarding patient navigation implementation mapped to CFIR^1^ constructs.

CFIR Construct	Exemplary Interview Quotation	Corresponding Fig 3 Theme
**Intervention Characteristics**		
Relative Advantage	“There is only like so much that…the medical team…the social service team can do…Navigation is more thorough, and we work more closely with the patient…If they were to meet [with] a social worker…[for]. . .housing [insecurity], the social worker may provide a housing program, and you know, just good luck. But with navigation we provide it and then we follow up, like, Were you successful?. . .Do you need assistance with application? Do you need to be connected with another program?”	High level of perceived relative advantage among navigators, care team members, and patients
Adaptability	“[Navigation] just depends what the patient’s needs are and what barriers each patient faces.”	High adaptability to patient need
Complexity	“…Keeping up with patient information [is challenging]. . .Being able to click on [a patient name], anyone can save notes,… whoever’s interacting with them from our team… without so [many]…little bits of communication needing to happen about a particular patient… being able to pull that up while you’re interacting with someone…would make things…easier…to manage so many people…”	High complexity of intervention
**Outer Setting**		
Needs/Resources of Those Served	““[Time] is most useful…understanding their needs and connecting them with the appropriate resources…it takes a little bit of time to…understand what resources they…qualify for or that would best suit them in their situations. And there’s always more resources coming up…[It’s important to] mak[e] sure that we are aware of resources and aware of what our patients need.”	High patient need level
Cosmopolitanism	“[A navigator is] a person. . .who’s. . .a bridge between providers and resources. I consider myself someone to be in the middle of it all—the medical team and resources.”	Communication with outside organizations
**Inner Setting**		
Readiness for Implementation	“Since we’ve been here for over a year now…everyone knows who we are, so that’s very helpful…being around people seeing our faces, I think that triggers [the care team] to contact us if they know our patient…Where it’s challenging is in different areas where the staff is not very familiar with us…”	Challenges with unfamiliar care teams
Implementation Climate	“I connect with patients and I don’t hear from them until 2…or 3 days later…If they don’t….text…or call me back…when I check in…that makes it harder for us to interact”	Varying levels of patient engagement
Networks and Communications	“I think it helps when we…message them [using the EMR] because it shows, ‘oh they have an [EMR] account…’ It would be more difficult if I was to call, like, ‘Who are you? Am I able to give you information?’…Through [the EMR], it shows…I’m part of the system…and they are more willing to help than if I was just to make a phone call.”	EMR facilitation of communication
**Characteristics of Individuals**		
Knowledge and Beliefs About the Intervention	“I sometimes question what patients would do if they didn’t have a navigator…I’m busy with tasks. So I wonder if patients didn’t have a navigator, how would they have done this? So I think it’s very beneficial [for] patients to have a navigator.”	All subthemes of Individual Characteristics
Self-efficacy	“I feel more integrated into the patient’s care…I am more confident in calling up the doctor or emailing the doctor, finding a way to connect…As to before I would kind of hold back…now it’s easier to pick up the phone [and] find a way to connect…”	Navigator self- efficacy
Individual State of Change	“Before, I was lacking the confidence of putting myself in the patient’s circle of providers…But [now] just being assertive in my role, like ‘Hey I’m her patient navigator, this is what I’m going to do…’”	Personal growth
Other Personal Attributes	“Communication…is…very important to master…If I don’t communicate properly…I might not be able to engage my patients or give a clear message to someone, whether it’s a nurse or a doctor I’m working with.”	Communication skills
**Process**		
Planning	“The training…exposed me to. . .people that I was going to interact with…I. . .had prior knowledge about general healthcare, [but] it really was helpful to know the specifics at [the institution]. . .The training was set for us. . .to meet different people…That was helpful to be able to learn more about their role and see how I was going to interact with them.”	Comprehensive training period
Reflecting and Evaluating	“I could really use more…motivational interviewing…I definitely have a few patients…they lack the motivation to engage in health…when they very clearly need it. . .I feel like if I knew how…I can best approach a situation like that…I wish I had more knowledge.”	Consistent reflection and evaluation of practice

Excerpt quotations from interviews with navigators (N = 3) conducted every 3 months. Quotations illustrate constructs within the Consolidated Framework for Implementation Research, demonstrating facilitators to and barriers of navigation implementation.

1. CFIR, Consolidated Framework for Implementation Research

### Intervention characteristics

Navigators consistently reported they perceived navigation as having a high *relative advantage* as compared to usual postpartum care. Per navigators, patient navigation takes usual care a step further to ensure patient access to regular, quality care. They felt navigation helped reduce patient responsibilities and improved engagement with care by “bridging the gap between the healthcare providers and the patient” to make sure needs do not “fall through the cracks.” Navigators also reported that both care team members and patients shared this positive perception: “I was able to stay for an appointment…go in…with the patient, introduce myself to the doctor…talk about a transition plan…they’re very appreciative of the information.”

A main perceived contributor to the positive relative advantage of navigation was the high *adaptability* to patient needs. Navigators emphasized the individualized nature of the intervention that allowed participants to receive support that directly targeted their personal barriers and needs. In describing her activities, one navigator noted, “It just depends on the patient…Once you get to know your patients, you’ll know [what they need]…” This variability both day-to-day and patient-to-patient facilitated personalized attention to participants’ barriers to care.

Such unpredictability carries a high degree of *complexity*, however, and navigators expressed this characteristic challenged their ability to support participants. More specifically, navigators reported challenges keeping up with variable participant needs and information, despite the standardized weekly log. One navigator emphasized, “It’s really hard to keep some sort of structure…because it’s just so different every day.” The wide variability of tasks and interactions with individuals including the participant, care team members, and community organizations complicated implementation. Further, the variety of needs prevented navigators from fully anticipating their workload, as “every day brings new situations, new challenges.” However, the sense of complexity evolved over time, becoming less prominent as expertise grew.

### Outer setting

Outer setting analysis of patient navigation revealed that the innovation successfully anticipated and met the *needs of those served*. Navigators reported that consistent, individualized interactions facilitated well-developed relationships with participants and a thorough understanding of their unique social, behavioral, and structural barriers to care. One navigator cited a participant who:

“Missed a few of her appointments because she didn’t have childcare…and the doctor was like, ‘we can’t see her anymore.’ And I reached out…and advocated on behalf of the patient and told them everything she was going through, and they [rescheduled her].”

The navigators perceived that attention to individual barriers thus successfully improved participants’ connection to the care system. Additionally, navigators reported connecting participants to community resources outside the health system, including diaper drives, furniture banks, and food pantries in their individual neighborhoods.

Such individualization also contributed to the navigation’s high degree of *cosmopolitanism*, the degree to which an organization or innovation is networked with other external organizations. The multi-level nature of the innovation motivated navigators to interact with individuals, clinics, and community organizations within and outside of the healthcare system. Initially, navigators expressed that unfamiliarity with outside organizations’ protocols challenged their ability to connect participants to resources. In the first interview, one navigator reported her inability to refer a participant to an organization because “as navigators and a research team…we couldn’t refer [patients].” Unfamiliarity with navigators’ role led to some organizations’ reluctance to share participants’ personal information with navigators. However, navigators’ increased familiarity with responsive individuals and community organizations throughout the postpartum year facilitated smooth implementation over time: “I try to network a lot…that’s been very helpful, staying connected with individuals that are experts in that department.”

### Inner setting

Inner setting analysis revealed the successful integration of navigators into the care team over time, implying the system’s *readiness for implementation*. Navigators reported that, in the early phase of implementation, care team members were reluctant to integrate navigators into patient care due to unfamiliarity with the navigation role and a desire for “the patient to be the ones to reach out.” However, as navigators worked consistently with clinic staff and developed relationships with care team members, integration became much more successful. One navigator recounted, “…after an appointment…[the doctor] came up to me and reviewed everything they had discussed with the patient and they said, ‘I told the patient if she needed help…to reach out to you.’” This partnership between navigators and care team members also facilitated care transitions, as navigators reported creating “a directory in my head of all the doctors that are available and [can accept] patients in the future.” Increased familiarization and relationship-building between navigators and care team members thus enhanced navigation’s implementation.

Such familiarity specifically improved the *implementation climate* throughout the postpartum year. In early stages, navigators reported feeling as though “the providers already have their own agenda.” Yet, ongoing communication and relationship-building with care team members, along with care teams’ increasing familiarity with the role of navigation, fostered improved climate. Additionally, *networks and communications* usage facilitated this evolution. Specifically, use of the electronic medical record fostered integration, as it “shows…we are part of the system.” Nevertheless, challenges persisted in interactions with outside health clinics. Some patients received primary care at offices that were not a part of the urban academic medical center; the personnel at these offices demonstrated hesitance to share patient information with navigators, due to an unfamiliarity with the navigator role. It was also difficult to access detailed information for these participants given these offices had distinct electronic medical records for ambulatory care. Navigators expressed they felt personnel at these clinics were less flexible with administrative operations and accommodating patient barriers and needs. Overall, navigators reported “it’s…more difficult to connect with their team…it causes delays in their care.” Nonetheless, navigators reported instances in which care from external clinics was still expedited due to navigation despite these logistical challenges.

Navigators also expressed challenges interacting with participants who did not readily engage in the innovation, as noted both in logs demonstrating multiple unsuccessful attempts at contact and navigator interviews. These participants either were self-sufficient in facilitating their own care, and thus felt they needed less navigation, or expressed less desire to interact with navigators. One navigator cited lack of engagement as the biggest challenge in maintaining a relationship with some participants, stating, “sometimes patients…are really busy or…have very low needs. So they don’t really reach out.” This experience demonstrates the importance of individualized navigation programming, as not all individuals, despite eligibility, will perceive similar need or desire for the intervention.

### Characteristics of individuals

Navigators possessed attributes that facilitated successful implementation over time. Soon after program initiation, one navigator reported, “I wasn’t really quite aware of what navigation meant…” As navigators gained experience, however, their *knowledge and beliefs* about navigation changed, as they expressed confidence in its utility and in their role as “the go-to person for patients.” Throughout the postpartum year, navigators demonstrated key personal attributes such as *self-efficacy*. Increased confidence in their own faculties and skills contributed to a positive *individual state of change*, improving the quality of the intervention. Navigators reported increased confidence reaching out to participants, care teams, and community organizations and “being [more] assertive in my role, letting them know…I’m a patient navigator…this is what I’m going to do.” These changes were reflected in navigators’ perceptions of improved patient engagement.

Navigators regularly emphasized the importance of quality communication skills, another key *personal attribute* facilitating implementation. Because they interact with a variety of individuals regarding complex patient situations, navigators highlighted the importance of “a clear message” to engage patients and care team members. Navigators reported communication with care team members, participants, and community organizations improved as they developed these skills.

### Process

In early interviews, navigators reflected on strengths and weaknesses regarding the *planning* of navigation, namely their training. They reported satisfaction with the immersive exposure to care team members and health systems practices throughout the training period “to get a full picture of everything I was going to encounter.” As lay individuals with limited prior healthcare experience, this interactive training, along with didactic training in navigation principles and health topics, was imperative to smooth implementation.

Interviews also gave navigators space for *reflecting on and evaluating* their navigation practices. In early interviews, navigators identified gaps in their training, specifically on the topic of motivational interviewing to engage participants with navigation or with specific health behaviors that had been recommended by the healthcare team. One navigator noted, “I could have…pushed in a more effective way to get them to open up and see what their needs were.” Over time and with continued training, however, navigators expressed increasingly positive perceptions of the innovation and its improved implementation within the health care system. Navigators described ongoing relationships with participants as “special to me,” and noted, “If I was ever part of a healthcare system, I would like to have a patient navigator.”

## Discussion

To our knowledge, no studies have used implementation frameworks to assess postpartum patient navigation in the United States. Assessing factors responsible for successful implementation is imperative to identify best practices and gaps that may inhibit its effectiveness or dissemination, even if it is shown to be efficacious in a trial setting. In the context of an ongoing RCT, we evaluated the implementation of postpartum navigation. Using the CFIR framework, we identified multiple features associated with successful implementation. Although a complex and fragmented healthcare system impedes patients’ ability to receive quality care, especially during the postpartum period, patient navigation offers an opportunity to support patients in an individualized manner during this period of transition [10]. Our assessment of implementation of postpartum patient navigation demonstrated its high utility for patients and care team members, as identified through activity tracking and interviews with navigators. Best practices such as electronic medical record usage, promoting familiarity with the navigation role, and consistent reflection on the innovation, which interviews promoted, all facilitated implementation.

The individualized nature of the innovation addressed patients’ unique needs at individual, systems-based, and community levels. Over time, the health system and care team members demonstrated a high readiness for implementation, as navigators used consistent communication and the electronic medical record to successfully integrate into the postpartum care team. Both systems-focused and personal development throughout the year facilitated navigation’s implementation, as navigators honed their skills. Importantly, however, the need for individualization contributed to a high degree of implementation complexity. Wide-ranging patient needs and levels of engagement, along with the fragmented healthcare and social services ecosystem, challenged the implementation of postpartum navigation yet also supported the rationale for the intervention. Complexity, varied engagement, and systems fragmentation all served as barriers to implementation. Navigator training and the support of care team members mitigated some of these challenges. These barriers and facilitators to implementation mirror those identified in existing literature that analyzes patient navigation in non-postpartum settings such as oncology [25–27]. Such concordance supports the use of implementation frameworks to identify barriers and facilitators to the implementation of postpartum patient navigation.

Optimized postpartum care requires the coordination of patient care with multiple care teams and community resources [28]. Systems constraints, differences in clinical practices, and fragmented transitions to long-term care make coordinated care challenging and exacerbate health disparities [29]. While implementation was overall successful, this analysis also demonstrates that not all individuals will find navigation desirable or necessary. For example, individuals who did not express needs beyond regularly scheduled navigator check-ins were less likely to consistently engage with navigators. These findings help elucidate potential sustainability and scalability practices for postpartum navigation programming.

Our findings also demonstrated the depth of navigation activities that are needed in the first year, as well as the wide range of interactions with both patients and care team members. Descriptive notes in log entries illustrated the complexity of navigators’ interactions with patients, as a single interaction usually covered multiple topics and one topic typically crossed multiple interactions. Our log and interview data suggest individuals with more complex needs and willingness to participate in healthcare are more likely to engage with and benefit from postpartum navigation. Further, log entries varied widely between patients, demonstrating the breadth and individuality of postpartum needs. Likewise, activities mirrored those completed by oncologic navigators, where navigation was first implemented, demonstrating consistent navigation practices across specialties [30–33].

Several specific features of the implementation context warrant mention. The site of study, a well-resourced urban academic medical center, may not represent the spectrum of postpartum care settings, and facilitators of and barriers to implementation may differ in other contexts. Further, this navigation program, occurring in the context of a clinical trial, included training and opportunities for continued education regarding navigation practices. Translating navigation from a research setting to sustainable long-term implementation will require additional facilitators, such as the financial and organizational support of health system administrators, who were not interviewed for this investigation. However, the knowledge this study provides regarding workload, complexity, and facilitators for navigation, along with future data regarding the innovation’s efficacy, can augment future cost-benefit analyses of postpartum patient navigation.

Despite the novelty of this analysis and its rich, layered use of real-time trial data, there are limitations to note. The relatively small sample of patients (N = 50) and navigators (N = 3) means the complete variability in navigator tasks may not have been appreciated. However, thematic saturation in interview and log data occurred, and consistent quantitative patterns of log data emerged. Additionally, it is possible that despite tracking their activities, the complexity and scope of the innovation may have caused navigators to miss documentation of interactions or tasks throughout the postpartum year. These omissions, however, would only underestimate the need for and breadth of navigation. Moreover, standardized training, programmatic goals and check-ins, and documentation logs likely limited such omissions or variability between navigators. Assessing implementation through the lens of navigators may also introduce bias, as individuals are likely to express pride in and highlight the successes of their work. Likewise, navigator interviews were not anonymous, potentially introducing social desirability bias. Nevertheless, positive attitudes about navigation came not only from navigators themselves but were also relayed to navigators by participants and care team members. Future investigation into the opinions of patients and care team members would further supplement implementation analysis. Finally, our data demonstrated changes in navigation implementation that occurred with increasing experience; this evolution of implementation suggests stories and experiences may differ for future participants.

## Conclusions

Assessment of the implementation of postpartum navigation at an urban academic medical center demonstrated facilitators of and barriers to successful integration of navigation into the health system. The frequent use of navigation by postpartum individuals as well as their care team members indicates its potential to optimize timely, comprehensive, and quality care for individuals with low income or other barriers to care. As patient navigation may be a promising method to optimize postpartum care, the ongoing study of its implementation, alongside study of efficacy, is essential to a full understanding of how to reduce maternal and neonatal health outcomes disparities.

## Supporting information

S1 Appendix Semi-structured interview guide. (DOCX)

S1 Table Mode of communication for navigator interactions with participants. (DOCX)
