# Supplementary material for: Implementation of postpartum navigation for low-income individuals at an urban academic medical center
Source: PLoS One. 2023 Feb 23;18(2):e0282048. doi: 10.1371/journal.pone.0282048 (PMC9949671; doi:10.1371/journal.pone.0282048)
Supplement: S1 Appendix — (DOCX) [file pone.0282048.s001.docx]

**Training**

- What experiences prior to being hired do you feel made you equipped to be a patient navigator?
- How would you describe the training you completed when you started as a patient navigator?
- After you began interacting with patients, did you face any unexpected issues or challenges?
  - Can you tell me a specific story of an unexpected challenge in your initial weeks as a navigator?
- How do you feel your training could have better prepared you for your role as a navigator?

**General Questions**

- How would you define your role as a patient navigator?
- What are the most common services you provide for patients?
  - Could you share a specific example where you assisted a patient in this way?
- Can you describe your average day as a patient navigator?
- How would you describe the differences between a patient navigator and a social worker?
  - Can you recall any instances in which you felt your role was markedly different from a social worker?
- What are the differences in your role as a navigator for a patient in the hospital versus a patient not in the hospital?
- How do you document your experiences as a patient navigator?

**Relationship with Patients**

- How would you describe your initial encounters with patients?
  - How do these encounters differ in person versus over the phone?
  - How do these encounters differ when patients are still pregnant versus postpartum?
- What are some of the effective ways you have developed a relationship with patients?
  - Could you recount a time where you felt you made strides in developing a relationship with a patient?
- What strategies do you use to build trust with your patients?
  - Can you share an example of a time you developed trust with a patient?
- What are the biggest challenges to developing relationships with patients?
  - Can you tell me a specific example in which you found it challenging to develop a relationship with a patient?
- How have your relationships with patients evolved over the postpartum period?
  - Can you share an example of a relationship with a patient that has evolved?
- Can you tell me about a time you became more disconnected from a patient as time passed?
  - Why do you think this happened?
  - What steps did you take to address the changes in your relationship?
- How have patients’ needs shifted throughout the postpartum period?
  - Can you share an example of a patient whose needs have shifted throughout the postpartum period?
  - How have you adapted to that shift?
- How did the COVID-19 pandemic affect the needs of your patients?
- How did the COVID-19 pandemic affect your relationship with patients?
  - What were the most notable changes in your interactions with patients?

**Relationship with Care Team**

- How do you feel about your integration into patients’ postpartum care team?
  - Can you share a time you felt integrated into patient care?
  - Can you share a time you felt like an outsider in the patient care team?
- How does your role as a patient navigator connect the patient to their care team?
- Can you describe your communication with the patient’s care team?
- How would you describe the relationship between yourself, the patient, and the patient’s physician?
  - Can you tell me a specific example of interactions between yourself, the patient and the physician which illustrate this relationship?
- How would you describe your relationship with patients’ nurses?
  - Can you tell me a specific example of an interaction which illustrates this relationship?
- In what instances have you felt you needed to direct a patient to a social worker, rather than assist them yourself?
  - Can you share a specific example of a time you connected a patient to a social worker?
- How does your relationship with the care team change, if at all, when your patient is in the hospital?
  - Could you share an example that illustrates this relationship?
- How does your relationship differ between yourself and the patient’s obstetric team versus other care teams (ie. primary care, specialists)?
- What are the biggest challenges you face in working with patients’ care team?
  - Can you tell me about a specific time you experienced this challenge?
- How did the COVID-19 pandemic affect your relationship with the healthcare team?

**Gaps in the Healthcare System**

- What barriers to accessing care have you witnessed your patients experience? By accessing her care, I mean transportation to care, insurance issues, and issues making appointments.
  - Can you share a time a patient faced a barrier to accessing her care?
  - How does your role as a navigator help eliminate these barriers?
- What challenges do you witness patients experience in their postpartum care? Examples of challenges may include lack of health literacy, health education, miscommunication with physicians, and self-advocacy issues etc.
  - Could you tell me a specific story in the last three months where a patient experienced challenges in her healthcare?
  - How do you feel navigation addresses these challenges?

**Personal Reflection**

- What do you like most about being a patient navigator?
- Where do you feel your time is most useful as a patient navigator?
  - Can you tell me about a time you felt useful in a patient’s postpartum care?
- Where do you feel you have spent time that hasn’t been useful for patients?
  - Can you tell me about a time you felt less useful for patients?
- How does being a patient navigator figure into your future career in healthcare?
- What unmet needs do you currently have as a patient navigator?
  - Can you tell me about a time a patient needed assistance that you could not provide her due to this deficiency?

Do you have any other thoughts or stories you would like to share which you feel describe your role as a navigator or the benefits of patient navigation?
