# Supplementary material for: Implementation of postpartum navigation for low-income individuals at an urban academic medical center
Source: PLoS One. 2023 Feb 23;18(2):e0282048. doi: 10.1371/journal.pone.0282048 (PMC9949671; doi:10.1371/journal.pone.0282048)
Supplement: S1 Table — (DOCX) [file pone.0282048.s002.docx]

| **% of Interactions Per Mode** | **Postpartum Month** | | | | | | | | | | | | |
| --- | --- | --- | --- | --- | --- | --- | --- | --- | --- | --- | --- | --- | --- |
|  | 1 | 2 | 3 | 4 | 5 | 6 | 7 | 8 | 9 | 10 | 11 | 12 | 13 |
| Face-to-Face | 12.2 | 4.1 | 0.9 | 1.7 | 3.7 | 2.0 | 2.7 | 3.6 | 0.0 | 3.1 | 6.3 | 2.2 | 5.4 |
| Text | 64.1 | 64.2 | 74.1 | 80.1 | 79.1 | 78.5 | 82.0 | 78.3 | 82.7 | 80.0 | 83.6 | 78.4 | 63.3 |
| Phone | 9.9 | 17.1 | 13.3 | 10.1 | 8.4 | 10.1 | 8.7 | 7.2 | 9.5 | 7.7 | 5.5 | 6.5 | 11.1 |
| Voicemail | 4.3 | 3.3 | 3.1 | 1.7 | 2.8 | 4.0 | 1.3 | 1.8 | 0.0 | 0.0 | 0.0 | 1.4 | 3.0 |
| Email | 4.9 | 5.5 | 3.7 | 2.0 | 1.4 | 1.3 | 2.0 | 7.2 | 1.6 | 6.2 | 2.3 | 5.8 | 8.4 |
| No Answer^1^ | 4.5 | 5.7 | 4.9 | 4.4 | 4.7 | 4.0 | 3.3 | 1.8 | 6.3 | 3.1 | 2.3 | 5.8 | 8.8 |

Percentage of navigator (N=3) interactions with patient participants (N=50) using each mode of communication

1. Indicates navigator attempted to call participant, but there was no answer and navigator did not leave a voicemail
